# Photonic Bandgap Propagation in All-Solid Chalcogenide Microstructured Optical Fibers

**DOI:** 10.3390/ma7096120

**Published:** 2014-08-26

**Authors:** Celine Caillaud, Gilles Renversez, Laurent Brilland, David Mechin, Laurent Calvez, Jean-Luc Adam, Johann Troles

**Affiliations:** 1 Glasses and Ceramics Group, Institut des Sciences Chimiques de Rennes, University of Rennes 1, 35042 Rennes Cedex, France; E-Mails: celine.caillaud@univ-rennes1.fr (C.C.); laurent.calvez@univ-rennes1.fr (L.C.); jean-luc-adam@univ-rennes1.fr (J.-L.A.); 2 Aix-Marseille Université, CNRS, Centrale Marseille, Institut Fresnel UMR 7249, 13013 Marseille, France; E-Mail: gilles.renversez@fresnel.fr; 3 PERFOS, Platform of Photonics Bretagne, 22300 Lannion, France; E-Mails: lbrilland@perfos.com (L.B.); dmechin@photonics-bretagne.com (D.M.)

**Keywords:** chalcogenide glasses, infrared fibers, microstructured optical fibers (MOFs), photonic bandgap fibers

## Abstract

An original way to obtain fibers with special chromatic dispersion and single-mode behavior is to consider microstructured optical fibers (MOFs). These fibers present unique optical properties thanks to the high degree of freedom in the design of their geometrical structure. In this study, the first all-solid all-chalcogenide MOFs exhibiting photonic bandgap transmission have been achieved and optically characterized. The fibers are made of an As_38_Se_62_ matrix, with inclusions of Te_20_As_30_Se_50_ glass that shows a higher refractive index (*n* = 2.9). In those fibers, several transmission bands have been observed in mid infrared depending on the geometry. In addition, for the first time, propagation by photonic bandgap effect in an all-chalcogenide MOF has been observed at 3.39 µm, 9.3 µm, and 10.6 µm. The numerical simulations based on the optogeometric properties of the fibers agree well with the experimental characterizations.

## 1. Introduction

Bandgap microstructured optical fibers (MOFs) also called photonic crystal fibers (PCFs) are often seen as the paradigm of MOFs [1]. The capability to guide light in a low index core surrounded by a higher average index microstructure exhibiting a forbidden propagation bandgap [2] is with no doubt the most intriguing properties of MOFs together with the endlessly single mode behavior of solid core conventional MOFs [3,4]. Numerous works both theoretical and experimental have already been dedicated to these low index core hollow core PCFs [5]. Nevertheless, only very few of them deals with high-index glasses like chalcogenide ones due to the difficulty of drawing such kind of vitreous materials [6,7,8].

Concerning bandgap guidance in chalcogenide MOFs, theoretical works have already been published but the associated experimental realization did not reach the targeted parameters preventing guiding in the hollow core [9].

In this article, we choose another route to reach bandgap guidance in chalcogenide MOFs: an all-solid MOF. All-solid chalcogenide MOFs have already been fabricated: in a first report, a multimode single ring AsSe/GeAsSe MOF was obtained [10]. In a second and more recent report, an all-chalcogenide MOF has been drawn using As_38_Se_62_ glass composition for the matrix and three rings of As_40_S_60_ (As_2_S_3_) inclusions [11]. It is worth mentioning that hybrid As_2_S_3_/tellurite and As_2_S_3_/As_3_Se_3_ MOF have also been obtained but in this case the fibers are not all-solid fibers [12,13].

To obtain bandgap guidance in an all-chalcogenide MOF, one must consider high index inclusions embedded in a low-index matrix (see [1] and references within). Such configuration is also known under the acronym ARROW for Anti Resonant Reflecting Optical Waveguides [14] even if, in this case, the emphasis is given to the property of the individual scatterers with high refractive index and not to the collective effect building the bandgap [15,16]. One can notice that bandgap guidance in near infrared has been obtained in a silica/chalcogenide configuration. In this case, the light propagation is still in the silica core [17].

Using numerical simulations, we have designed an all-solid bandgap MOFs made of an As_38_Se_62_ matrix with high-index Te-As-Se glass (TAS) inclusions. Then, the designed MOFs have been fabricated, and characterized in terms of their transmission spectra in the near and mid infrared. Core guidance was investigated at several wavelengths up to 9.3 µm.

## 2. Fabrication of the Fibers

### 2.1. Glass Synthesis and Characteristics

**Figure 1:**
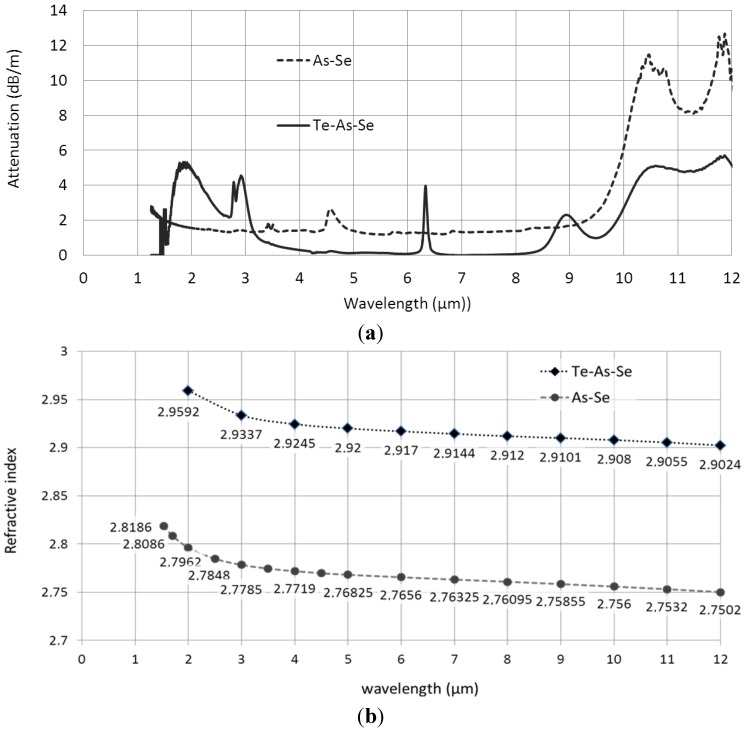
(**a**) As_38_Se_62_ and Te_20_As_30_Se_50_ glasses attenuation curves; and (**b**) refractive indices of As_38_Se_62_ and Te_20_As_30_Se_50_ glasses.

Before the elaboration of MOFs, two glass compositions have to be elaborated: As_38_Se_62_ and Te_20_As_30_Se_50_. The glass rods were synthesized by melting, in a rocking furnace, high purity arsenic, selenium and tellurium at 850 °C in a silica ampoule under vacuum (1 × 10^−4^ mbar). The melts are then quenched under water and annealed around the glass transition temperature *T*_g_ (*T*_g_ = 165 °C and 137 °C for As_38_Se_62_ and Te_20_As_30_Se_50_, respectively). Other specific treatments using oxygen and hydrogen getters (aluminum metal and tellurium tetrachloride, respectively) associated with successive distillations are necessary to remove impurities such as water, oxygen, hydrogen and carbon [18,19]. The attenuation curves of the two glasses used for the elaboration of the MOFs are given in Figure 1. The lowest attenuation is less than 2 dB/m in the 5–9 µm spectral range for As-Se glass and less than 0.5 dB/m in the 4–6 µm and 7–8 µm spectral domains for Te-As-Se based glass. The low refractive index glass matrix is As_38_Se_62_, with indices varying between 2.82 and 2.75 in the mid-infrared. The high-index inclusions are made with Te_20_As_30_Se_50_ glass, whose refractive index is in the range of 2.96–2.90, depending on the infrared wavelength. Wavelength-dependence of the refractive indices is given in Figure 1b for the two glass compositions.

### 2.2. Microstructured Fiber Fabrication

**Figure 2:**
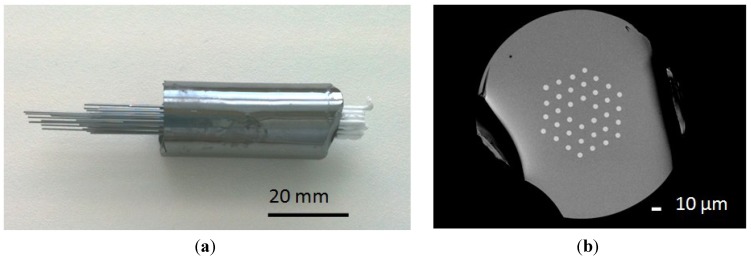
(**a**) As_38_Se_62_ and Te_20_As_30_Se_50_ preform; and (**b**) scanning electron microscope (SEM) image of Fiber 1 cross section (backscattering electrons image).

The As_38_Se_62_/Te_20_As_30_Se_50_ microstructured fiber was obtained by using a three-step process. First, an As_38_Se_62_ microstructured preform was elaborated by the molding technique, as described in [20]. The mold contains silica capillaries threaded into silica hexagonal guides in a silica ampoule [20]. An As_38_Se_62_ glass rod is placed at the top of the mold and is then heated to become almost liquid. It must be soft enough to flow down in the silica mold. Once the glass is in the mold, the tube is quenched in air and annealed at *T*_g_. Finally, the silica capillaries embedded in the preform are removed by applying a hydrofluoridric acid solution. The outer diameter of the preform is 20 mm and the holes diameters are around 500 µm. Then, a Te_20_As_30_Se_50_ rod with an outer diameter of 12 mm was drawn into a 470-µm diameter fiber. The hybrid preform is then obtained by the insertion of Te-As-Se fibers into the 36 holes of the As-Se molded preform, as shown in Figure 2a. Finally, the composite preform was drawn into fibers with three different outer diameters (230, 200 and 165 µm) in order to obtain different final geometries and different propagation properties. Figure 2b shows an example of the geometry obtained for Fiber 1.

The geometrical parameters of the different fibers (Fibers 1, 2 and 3) are given in Table 1. The three fibers present nearly the same *d*/Λ ratio (between 0.364 and 0.377). However, a higher variation is observed for Fiber 3, which could be due to the drawing process and/or the low resolution of the backscattering scanning electron microscope (SEM) pictures.

**Table 1:** Geometrical parameters of the fibers.

Fiber	Outer diameter	*d* (µm) ± 0.1 µm	Λ (µm) ± 0.1 µm	*d*/Λ ± 0.01	Core diameter
1	230 µm	5.5	14.7	0.374	23.9
2	200 µm	4.9	13.0	0.377	22.3
3	165 µm	4.3	11.8	0.364	19.4

## 3. Results

### 3.1. Experimental Transmission of the Fibers

**Figure 3:**
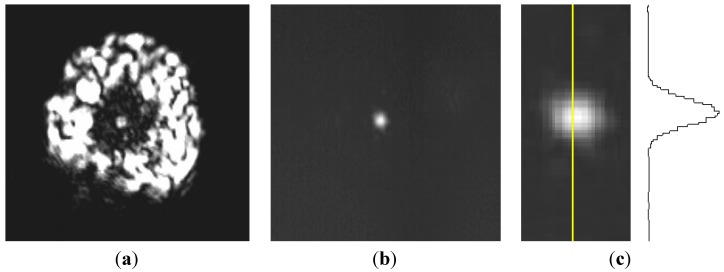
Near field observation at 3.39 µm of the Fiber 1: (**a**) without Ga-Sn coating; (**b**) with Ga-Sn coating in order to remove the cladding mode; and (**c**) Gaussian profile of the photonic bandgap fiber core.

Optical transmission of the fibers was measured with a Bruker Fourier Transform Infrared Spectroscopy (FTIR) (Billerica, MA, USA). The black body light of the FTIR is injected into a 40 cm-long fiber and the signal is detected with a nitrogen-cooled mercury, cadmium, tellurium (MCT) detector. Due to the large size of the injected spot, the light is not injected only in the core of the fiber. Indeed, part of the infrared spectroscopy (IR) light is injected in the clad of the fibers and propagates in the clad. In order to detect only the light that propagates in the core, a high-refractive-index Ga-Sn alloy is applied on the surface of the fiber in order to remove the cladding modes. Indeed, the Ga-Sn alloy presents a very weak reflection coefficient together with high loss which leads to an absorption of the cladding light after only few centimeters (see Figure 3a,b).

**Figure 4:**
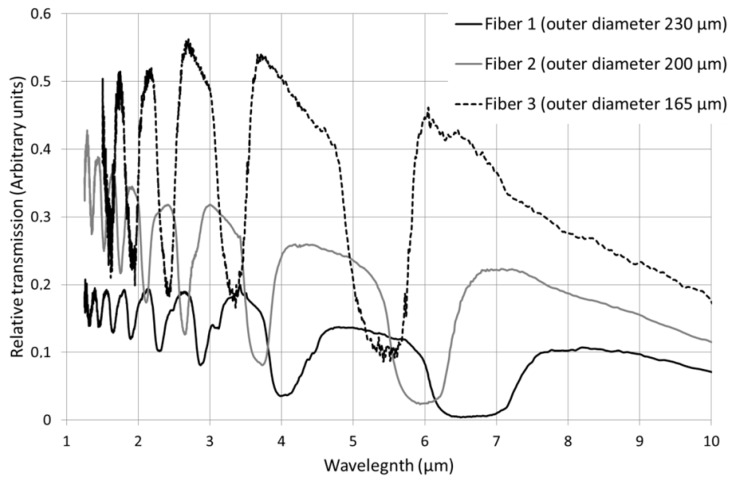
Infrared transmission bands of the Fibers 1, 2 and 3.

Figure 4 shows the transmission bands of the three fibers recorded after removing the contribution of the cladding mode. In all the fibers, transmission bands have been brought out in the 1.5–10 µm infrared range. It is worth noting that transmission curves are given in arbitrary units, so intensity of transmission bands cannot be compared. However, reliable information can be obtained by examination of the position of transmission bands (Figure 4), in accordance with the calculations presented in Section 3.3 that predict the position of transmitting and non-transmitting spectral domains.

### 3.2. Near-Field Measurements

Light propagation was demonstrated for monochromatic light at 3.39 µm from a He-Ne laser injected, as an example, in Fiber 1 (larger core). The near-field image at the output of the fiber was visualized on an infrared camera (FLIR PM 390, FLIR Systems, Wilsonville, OR, USA). Due to the low power of the laser and significant optical losses of the core of the fiber, near-field measurements have been carried out for a 4 cm-long fiber. Indeed, the experimental optical losses of the core were estimated to be in the 20–50 dB/m range. Figure 3 shows the near-field image obtained in a 4 cm-long sample of Fiber 1. The first near-field capture (Figure 3a) is observed when the light propagates in the core and the clad of the fiber. The second near-field image (Figure 3b) is obtained when a Ga-Sn alloy is applied at the surface of the fiber in order to remove the cladding modes. In this case, a Gaussian profile is observed in the core localized mode of the fiber (Figure 3c).

**Figure 5:**
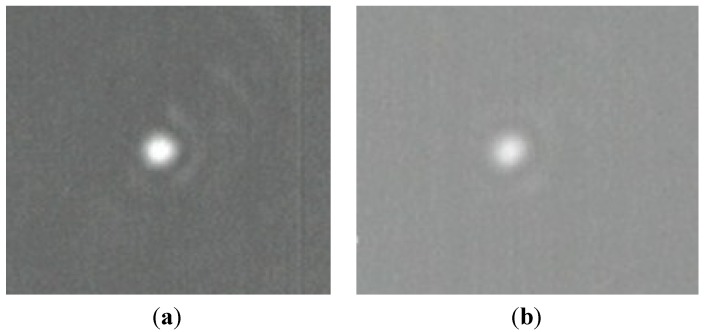
Near field observation of the Fiber 1 at: (**a**) 9.3 µm; and (**b**) 10.6 µm.

In a second step, near-field measurements were performed on the same fiber by using a tunable CO_2_ laser. Figure 5 shows the intensity profile at the output of Fiber 1 at 9.3 µm and 10.6 µm. For those wavelengths, intensity profiles were recorded with a FLIR E320 infrared camera (FLIR Systems).

### 3.3. Modeling

The theoretical guiding properties of the fabricated microstructured fibers have been obtained using the multipole method [21]. In the simulations, the fiber geometrical parameters and the material dispersion properties are taken into account [7].

**Figure 6:**
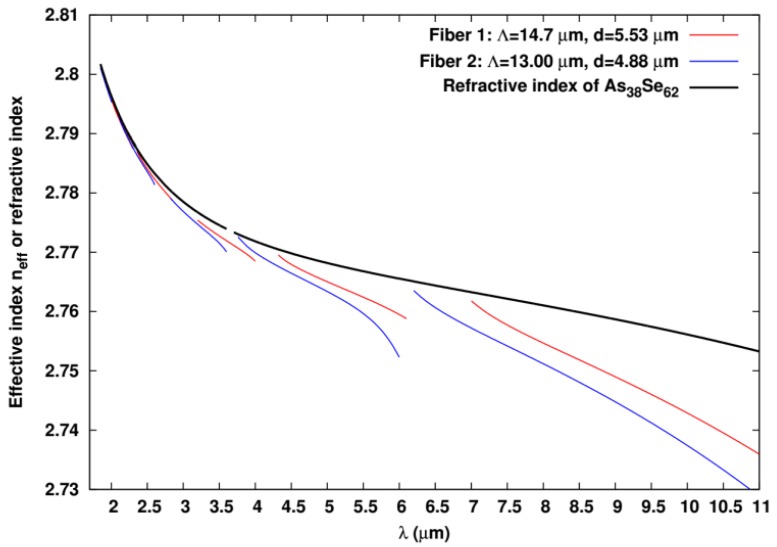
Material dispersion for the matrix As_38_Se_62_ used in the numerical simulations and computed effective index of the fundamental core localized mode for Fibers 1 and 2 as a function of the wavelength.

The fiber geometrical parameters are given in Table 1. Fibers are made of *Nr* = 3 rings of high-index Te_20_As_30_Se_50_ inclusions embedded in the low-index As_38_Se_62_ glass. In Figure 6, the material dispersion curve for the As_38_Se_62_ glass is shown. Due to the ARROW guiding mechanism, a bandgap-like effective index dispersion is observed for the effective index of the fundamental core-localized mode of modelized Fibers 1 and 2. This behavior is characterized by successive effective index dispersion curves with small slope terminated at both low and high wavelength extremities by high slope regions associated to the delocalization of the fundamental mode in the high index inclusions of the optical cladding microstructure ([1] and see references inside). The high-slope regions are associated to the high guiding loss region. As expected for core localized mode, the effective index of the small slope regions is below the refractive index of the As_38_Se_62_ glass.

**Figure 7:**
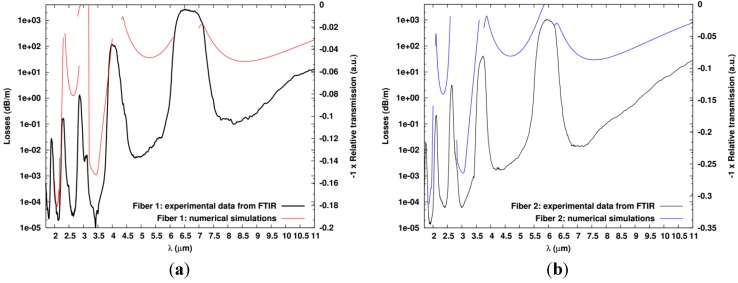
Computed guiding losses (without taking into account any material losses) in dB/m with a log-scale (left *y*-axis) and sign-reverse of the measured relative transmission in arbitrary unit (right *y*-axis) *vs.* the wavelength: for the (**a**) Fiber 1; and (**b**) Fiber 2.

As it can be seen in Figure 7a, where both the computed guiding losses for Fiber 1 and the sign-reverse of the measured relative transmission for the same fiber *versus* the wavelength are provided, the computed results for both the low-loss and high-loss regions fit nicely with the experimental data. All the bandgaps are recovered in all the studied wavelength range. The non-monotonous behavior of the loss minima as a function of bandgap order, as seen in the computed results in Figure 7a, is a known property of ARROW MOF [1,16].

In Figure 7b, similar results as the one shown in Figure 7a are given but for Fiber 2. Once again, positions of the computed bandgaps agree nicely with the measurements. Comparing the results for Fiber 2 with the ones computed for Fiber 1 shows that, as expected, for a fixed *d*/pitch ratio, the wavelengths of the transmission bands scale nearly linearly with the pitch since Maxwell equations are scale invariant and since the material dispersions of the two glasses are not huge. Similar results are also obtained for Fiber 3 (data not shown). This confirms that the guiding mechanism occurring in the fabricated fibers is really the ARROW type one.

## 4. Discussion

A hybrid preform made with an As-Se matrix and high-index inclusions of TAS has been drawn. Three microstructured fibers with three different outer diameters were obtained. The transmissions of those three fibers are compared in Figure 2. Several transmission bands have been brought out in the mid-IR which is a typical signature of a photonic bandgap behavior. The positions of the bands vary in an expected way with the geometry of the fiber.

The agreement between the numerical simulations and the experimental data related to bandgap positions and core guidance observations are fairly good. Nevertheless, this is not the case for the loss values. Measured losses are in the 20–50 dB/m range at 3.39 µm while, according to the guiding losses computed without taking into account any material losses, they should be below 0.01 dB/m at this wavelength. Even if the material losses of the two raw glasses As_38_Se_62_ and Te_20_As_30_Se_50_ as described in Section 2 are taken into account in the simulations, the computed loss level does not reach the measured one. Consequently, other types of losses must be taken into account. One highly probable possibility is the existence of defects at the glass interface region between the As_38_Se_62_ matrix and the Te_20_As_30_Se_50_ inclusions. The defects can be crystals and/or small bubbles, as already demonstrated by Brilland *et al.* [22]. Such loss increase have already been observed in the first fabricated complex all-solid all-chalcogenide MOF [11] even if the loss gap (approximately 7 dB/m) between the computed losses and the measured ones was smaller than in the present case. As already observed in chalcogenide fibers, crystallization can occur during the drawing step, more particularly when several drawings are needed [19]. Such crystallization can induce strong additional optical losses by scattering. For the demonstration of the first photonic bandgap propagation in a chalcogenide fiber, the hybrid preform (As_38_Se_62_ and Te_20_As_30_Se_50_ glasses) was built neither under neutral atmosphere nor with controlled concentration of ambient dust. Consequently, in order to avoid the presence of additional losses due to the imperfect interfaces qualities or/and crystallization during the drawing, the glasses purification, the preform elaboration and the drawing operation have to be improved. This is a task for future work.

## 5. Conclusions

A hybrid preform made with As-Se matrix and high-index inclusions of TAS was drawn. Three microstructured fibers with three different outer diameters were obtained. Optical transmissions of those fibers have been measured from 1.85 µm up to 11 µm. Several transmission bands have been brought out, notably in the mid-IR, which is a typical signature of a photonic band gap behavior. The positions of the bands vary with the geometry of the fiber as expected from theoretical scaling argument. Guidance in the core was observed at 3.39 µm, 9.3 µm, and 10.6 µm. Numerical simulations taking into account the geometry and the material properties confirm the existence and the positions of the bandgaps, and also core guidance at the wavelengths utilized for the experiments. The next step is to reduce the overall losses of the fabricated MOFs by using improved drawing techniques.
